# Emerging open-channel droplet arrays for biosensing

**DOI:** 10.1093/nsr/nwad106

**Published:** 2023-04-20

**Authors:** Yongchao Song, Lirong Wang, Tailin Xu, Guangyao Zhang, Xueji Zhang

**Affiliations:** School of Biomedical Engineering, Health Science Center, Shenzhen University, Shenzhen 518060, China; Intelligent Wearable Engineering Research Center of Qingdao, Research Center for Intelligent and Wearable Technology, College of Textiles and Clothing, State Key Laboratory of Bio-Fibers and Eco-Textiles, Qingdao University, Qingdao 266071, China; School of Biomedical Engineering, Health Science Center, Shenzhen University, Shenzhen 518060, China; School of Biomedical Engineering, Health Science Center, Shenzhen University, Shenzhen 518060, China; Intelligent Wearable Engineering Research Center of Qingdao, Research Center for Intelligent and Wearable Technology, College of Textiles and Clothing, State Key Laboratory of Bio-Fibers and Eco-Textiles, Qingdao University, Qingdao 266071, China; School of Biomedical Engineering, Health Science Center, Shenzhen University, Shenzhen 518060, China

**Keywords:** open-channel droplet, wettable pattern, mini-pillar platform, liquid marbles, multiple biosensing

## Abstract

Open-channel droplet arrays have attracted much attention in the fields of biochemical analysis, biofluid monitoring, biomarker recognition and cell interactions, as they have advantages with regard to miniaturization, parallelization, high-throughput, simplicity and accessibility. Such droplet arrays not only improve the sensitivity and accuracy of a biosensor, but also do not require sophisticated equipment or tedious processes, showing great potential in next-generation miniaturized sensing platforms. This review summarizes typical examples of open-channel microdroplet arrays and focuses on diversified biosensing integrated with multiple signal-output approaches (fluorescence, colorimetric, surface-enhanced Raman scattering (SERS), electrochemical, etc.). The limitations and development prospects of open-channel droplet arrays in biosensing are also discussed with regard to the increasing demand for biosensors.

## Teaser

This review summarizes the typical representatives of open-channel microdroplet arrays and focuses on diversified biosensing integrated with multiple signal output approaches (fluorescence, colorimetric, SERS, electrochemical, etc.).

## INTRODUCTION

A droplet array can easily fulfill requirements of complex reaction and sequential process experiments owing to its advantages of miniaturization and versatility, which play an important role in a wide range of scientific and industrial applications, including synthesizing multifunctional materials, controlling interfacial reactions, surface rheology studies and digital microfluidic technology [1–5]. Micro-nanoliter droplets facilitate the study of rare biological and clinical samples, and also act as a desirable platform for surface reactions and interface assembly because of the larger specific surface area–volume ratio [6,7]. Furthermore, the droplets exhibit excellent mixing and mass transfer over shorter diffusion distances, thereby avoiding boundary effects, providing a large-scale quantitative response platform for high-throughput analysis [8]. Droplet microfluidics platforms have inherited the above advantages while achieving automation and high throughput by integrating complex micropump and microvalve systems, which block the droplets in the microfluidic channel or oil layer and prevent full gas–liquid interaction [9]. Superior to the enclosed droplet system, a physical or chemical well of open-channel droplets replaces the nonpolar solvent, in order to produce droplet separation and effectively avoid unnecessary solvent residue in a closed conduit. Direct droplet formation, fixation and movement by a pipette or dropper dispenses with the integrated complex micropump and microvalve when it comes to manipulating the discrete droplets for moving, mixing, splitting and other processes, which greatly simplifies the preliminary preparation of droplet application and expands applicable scenarios.

In recent years, enormous efforts have been dedicated to developing an open-channel droplet array in biosensing, which exhibits droplets anchoring physically or chemically well for subsequent droplet manipulation and analysis [10,11]. Meanwhile, an open-channel platform also provides unique advantages for improving the efficiency and accuracy of trace biosensing. Firstly, the volume of reaction in the droplet is as small as a microliter or nanoliters, which can greatly decrease the consumption of reagents. Secondly, individual droplets can perform parallel high-throughput analysis for multiple targets. Thirdly, microdroplets can accelerate biochemical reactions because of the rapid blend of reactants and high heat and mass transfer efficiency. Lastly, miniaturized reactors with an open system afford easy accessibility for purification, mixing and amplification, and allow for versatile combinations of various signal-output approaches.

**Figure 1.**
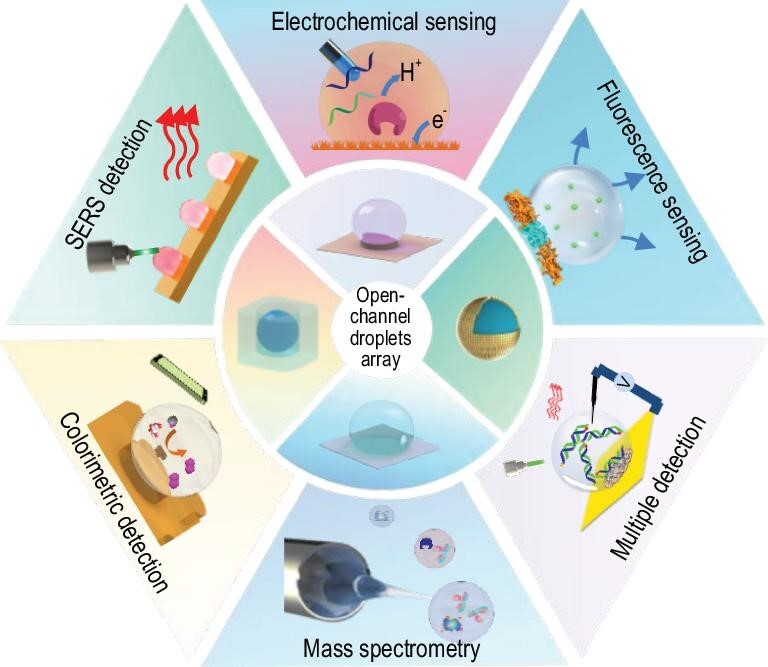
Open-channel microdroplet array toward multiple biosensing.

This review summarizes the recent progress of the emerging open-channel droplet array for multimode and multifunctional biosensing. The section entitled ‘Typical open-channel droplet platform’, mainly introduces the four typical open-channel droplet platforms (microchamber, wettable pattern, mini-pillar platform and liquid marbles) and their constraint methods, fabrication materials, advantages and inherent limitations in biosensing. The section entitled ‘Applications of emerging open-channel droplets’, highlights the recent progress of multiple signal acquisition techniques (fluorescence, colorimetric, SERS, electrochemical, etc.) that are coupled with an open-channel system for multiple biosensing, as shown in Fig. 1. At the end, we share personal viewpoints on the future research direction and remaining challenges of the current open-channel droplet platform with regard to its much-in-demand practical application.

## TYPICAL OPEN-CHANNEL DROPLET PLATFORM

The disposable and versatile open-channel droplet platform is a prerequisite for manipulating droplets for extensive biochemical sensing [12]. Here we summarize typical open-channel platforms for biosensing, and divide them into four cases based on the position relationship between the open-channel droplet and platform interface: (i) droplet under surface, where the contact interface of liquid and solid is lower than the surface of the platform, and physical outer walls trap the droplets to form the microchamber; (ii) droplet at surface, which combines different types of wettability (hydrophobicity, hydrophilicity) in one substrate to form a wettable droplets pattern; (iii) droplet on surface, where the contact interface of liquid and solid is higher than the surface of the platform, and the mini-pillar array above the surface forms the open-channel droplets by adhesion and physical boundary restriction; and (iv) droplet in surface, where the hydrophobic micro/nanoparticles surround the entire droplet to form an analogous soft solid named ‘liquid marbles’. As such, the loose layer of particles imprisons the droplets as individual reactors, and plays multiple roles such as SERS substrate or working electrode for multiple sensing [13]. Furthermore, the unique optical, electric, thermal and magnetic properties of particles promote the layer that opens/closes for optical analysis and other applications [14]. Each platform has its unique strategy for imprisoning and forming open-channel droplets, alongside its specific application advantages and inevitable internal restrictions, as shown in Table 1.

**Table 1.**
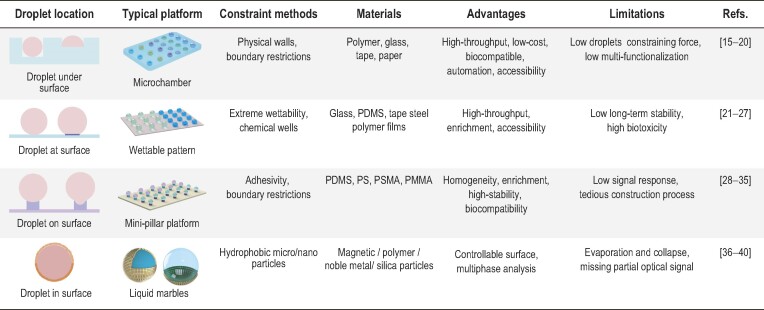
Summary of different typical open-channel platform features for biosensing.

## APPLICATIONS OF EMERGING OPEN-CHANNEL DROPLETS

The open-channel droplet array is widely used in biosensing and has the advantage of reduced sample and reagent consumption, offering the possibility of *in situ* analyses and increased sensitivity caused by faster diffusion at the microscale [12]. In particular, accessible manipulation of droplet anchoring, fusion, splitting and directional motion provide the possibility of multiphase interaction and multiplexed analysis. Evaporation of open-channel droplets is inevitable in multiple biosensing. To avoid undesired droplet evaporation, the relatively long reaction process in the droplet is usually completed under high humidity or oil layer protection. The evaporative enrichment required in the sensing process is usually achieved with ∼5–10 mins of natural evaporation in most techniques such as fluorescence, mass spectrometry (MS) and colorimetric detection. Some reports employ photothermal SERS substrate to heat up sampling and promote evaporation [41]. For electrochemical sensing, the droplets are necessary for the sensing process without the evaporative enrichment property, and droplet evaporation is negligible due to the rapid electrochemical signal response. In this section, we focus on such sensing applications of open-channel droplets by combining them with signal-output approaches (fluorescence, electrochemical, SERS, colorimetric, MS and multiple methods).

### Open-channel droplet array for fluorescence sensing

Fluorescence analysis is the most common technique with the advantages of high sensitivity and selectivity for diversity biosensing [42]. An open-channel droplet array can efficiently and homogeneously concentrate the fluorescent molecules in the millimeter/micrometer-level spots after evaporation enrichment to further improve sensitivity and accuracy, with a lowest limit of detection (LOD) of ∼10^−16^ M for fluorescence imaging and analysis [27,43]. Moreover, a high-density droplet array in one chip provides a rapid detection speed (<10 s) with high throughput and simultaneous biosensing, catering for commercial and portable applications [44,45]. The following sections will highlight the integration of fluorescence technology with open-channel droplet arrays for diverse biosensing.

**Figure 2.**
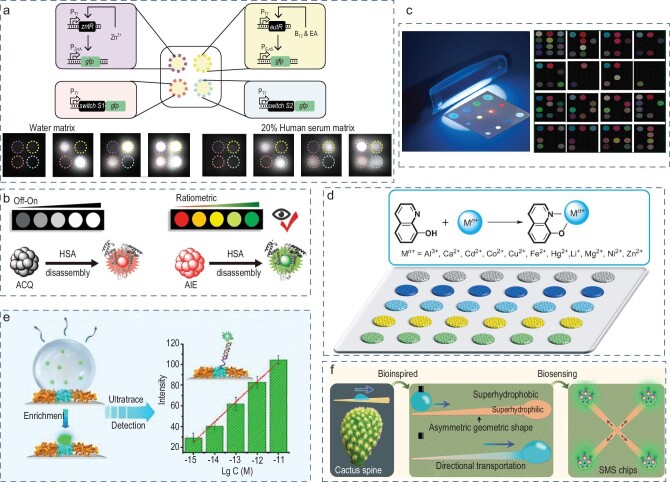
Open-channel droplet array for fluorescence sensing. (a) Protocell arrays for multiple clinically relevant biomarker sensing. Reproduced with permission from ref. [46]. Copyright 2021 Springer Nature. (b) Paper-based device toward human serum albumin texting. Reproduced with permission from ref. [15]. Copyright 2020 Wiley-VCH Verlag GmbH & Co. KGaA, Weinheim. (c) Biogenic amine detection on the paper-based sensor array. Reproduced with permission from ref. [47]. Copyright 2021 Elsevier. (d) Multiple metal-ion recognition on the photonic-crystal microchip. Reproduced with permission from ref. [48]. Copyright 2013 Wiley-VCH Verlag GmbH & Co. KGaA, Weinheim. (e) Superwettable wells toward trace DNA sensing. Reproduced with permission from ref. [49]. Copyright 2015 Wiley-VCH Verlag GmbH & Co. KGaA, Weinheim. (f) Open-channel droplet transportation for biosensing. Reproduced with permission from ref. [50]. Copyright 2020 American Chemical Society.

Simultaneous detection of multiple analytes on a single chip can guide complex decision-making without increasing the number of operations and devices. A customizable microchamber platform with a polymer-based aqueous two-phase system can directly and simultaneously perform four-target fluorescence sensing using the ChemiDoc MP imager, as demonstrated in Fig. 2a [46]. Reducing the need for professional and expensive fluorescence equipment, a paper-based chip, combined with a smartphone, achieved the multi-concentration detection of human serum albumin by comparing fluorescence intensity ratios, and realized a point-of-care test for blood samples (Fig. 2b) [15]. The integration of a droplet array promotes the development of a high-throughput platform for high-density droplet fluorescence detection. For example, the polyvinylidene fluoride (PVDF) membrane integrated four cycloplatinated (II) complexes and eight metal nanoclusters to achieve rapid discrimination and high-throughput fluorescence detection of biogenic amines, as shown in Fig. 2c [47]. Similarly, Huang *et al.* reported a photonic-crystals-assembled superwettable microchip for selective 12 metal ion fluorescence sensing (Fig. 2d) [48]. The microchip possesses six parallelizable channel spots with different diameters of photonic crystals, and readouts of the 72 results of the multiple ion samples based on the different enhancing or quenching effects of the fluorescence, which reveals the great importance of developing advanced fluorescent systems for complex samples analysis.

The evaporation of microdroplets, alongside condensation in designated microwells, increases the collision and recognition between the target and capture, thus providing a higher signal output. Superwettable microchips that have evaporation enrichment in order to ultratrace DNA fluorescent sensing were demonstrated in Fig. 2e [49]. The microwells enrich the targets in a highly diluted solution through a continuous evaporation process, and achieve ultrasensitive DNA fluorescent detection with a detection limit of 10^−16^ M, which is an improvement of 10 orders of magnitude over commercial hydrophilic glass. Subsequently, inspired by water-collecting cactus spines, the researchers designed a geometric asymmetric superhydrophilic structure on superhydrophobic nanomaterial-based chips for fluorescent sensing (Fig. 2f) [50]. The sample with a prostate-specific antigen was dropped at the inlet of the chip, dispersed to multiple channels and immobilized on prostate-specific antibody-modified microwells for sandwich fluorescence detection with a detection limit of 1.0 × 10^−12^ g mL^−1^, fulfilling the sensitivity requirements of clinical detection.

**Figure 3.**
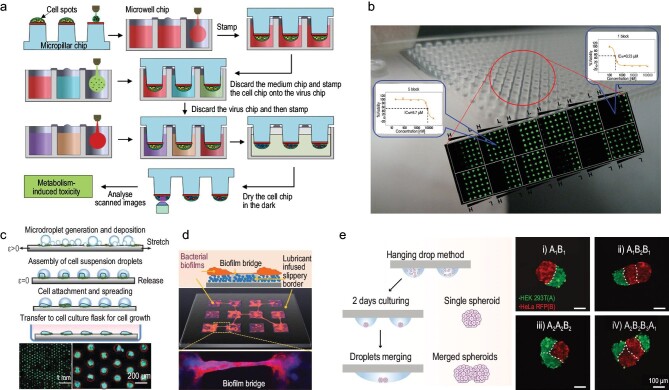
Fluorescence signal-based cell analysis. (a) Experimental procedure of the mini-pillar TeamChip for toxicology screening. Reproduced with permission from ref. [29]. Copyright 2014 Springer Nature. (b) Micropillar/microwell chip platform toward screening analysis of anticancer drug efficacy. Reproduced with permission from ref. [33]. Copyright 2014 American Chemical Society. (c) Surface-assembled microdroplets for facile production of cell arrays. Reproduced with permission from ref. [55]. Copyright 2020 Wiley-VCH Verlag GmbH & Co. KGaA, Weinheim. (d) Biofilm bridge formation on patterned lubricant-infused surfaces. Reproduced with permission from ref. [56]. Copyright 2019 Wiley-VCH Verlag GmbH & Co. KGaA, Weinheim. (e) Programmable merging of cell spheroids using the miniaturized droplet microarrays. Reproduced with permission from ref. [57]. Copyright 2021 Wiley-VCH Verlag GmbH & Co. KGaA, Weinheim.

The open-channel droplet platform realizes a high-throughput 2D/3D cell culture while easily realizing cell transfer and stimulating response, the merging of cells and information interaction [37,51,52]. Currently, cell-based analyses, including cell cultures, drug screening, gene expression and cell fusion interaction, are almost dependent on fluorescence signals [53,54]. A high-density cell array can directly generate a mini-pillar for fluorescence toxicology screening, as shown in Fig. 3a [29]. Similarly, an open-channel micropillar/microwell platform anchored the U251 brain cancer cell line and three primary brain cancer cells from patients, and tested the therapeutic effects of 24 anticancer drugs by measuring their dose-response (Fig. 3b) [33]. Furthermore, cell droplets can form a high-density array on the superwettable chip by dynamic self-assembly, as shown in Fig. 3c [55]. Cell-loaded aerosol droplets are generated by microfluidics and sputtered onto the surface of an elastomeric film with a superwettable pattern. By simple stretching and recovery, the cells can move away from the low-adhesion superhydrophobic surface toward the hydrophilic array for further cell culture and fluorescence detection.

Open-channel droplets can realize merging and interaction through ‘biofilm bridges’, which allow the individual bacteria suspension in hydrophilic spots to spread over bacteria-repellent lubricant-infused regions, as shown in Fig. 3d [56]. The Programmable Merging of Adjacent Droplets method (proMAD method) has realized the on-demand fusion of individual cell spheroids into the complex 3D cellular architecture by controlling the merging of two to six neighboring cell droplets (Fig. 3e) [57]. Long-range signaling in different cell populations demonstrates potential applications in diverse fields, ranging from basic biology to cancer research.

Fluorescence is one of the most common and mature technologies for biochemical molecules and cell sensing. Open-channel droplets are helpful for anchoring and enriching the sample in the process of fluorescence analysis, and have great potential in the screening of ultratrace biomarkers, and early diagnosis. However, droplet fluorescence imaging requires complex equipment such as fluorescence or confocal microscopes, which makes the simple and portable strategy of open-channel droplets meaningless. A future development direction would be to simplify the fluorescence equipment to match the miniaturization platform for portable and point-of-care biosensing.

### Electrochemical sensing in an open-channel droplet array

Electrochemical methods possess numerous complementary advantages in the field of biochemical analysis, including high sensitivity, high selectivity, and label-free and addressable sensing, whereas the bulk system of traditional electrochemical methods is difficult to realize in high-throughput sensing and rare samples analysis [58]. An open-channel electrochemical chip that integrates the integrated circuit system in a platform with an array of droplets successfully solves this problem, avoids the cross-contamination of droplets and achieves the simultaneous electrochemical analysis of independent and high-throughput nano/microliter droplets [59,60].

**Figure 4.**
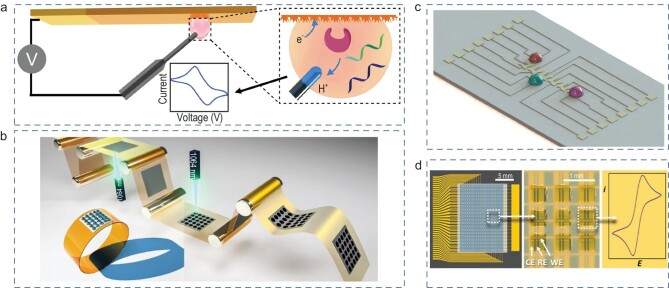
Electrochemical sensing in an array of open-channel droplets. (a) Superwettable chip for cancer-biomarker electrochemical sensing in an individual droplet. Reproduced with permission from ref. [61]. Copyright 2018 American Chemical Society. (b) The sweat capture device for glucose sensing. Reproduced with permission from ref. [62]. Copyright 2019 American Chemical Society. (c) Additively manufactured biosensor toward ion-selective detection. Reproduced with permission from ref. [63]. Copyright 2019 American Chemical Society. (d) High-density droplet array toward addressable electrochemical measurement. Reproduced with permission from ref. [64]. Copyright 2017 American Chemical Society.

The combination of open-channel interface and electrode array effectively anchors electrochemical detection in trace droplets. The superwettable nanodendritic electrochemical biosensor confined to the open-channel droplets in microwells employs a two-electrode system for multiple prostate-cancer biomarker sensing (Fig. 4a) [61]. A graphene-based superwettable sweat-capture device shows the concentration level of glucose in sweat, in order to non-invasively monitor diabetes in real time, as shown in Fig. 4b [62]. With the functionalization and integration, the chips are not limited to droplet anchoring, but attempt to manipulate and address the high-density droplets. The digital microfluidic sensor that manipulates the open-channel droplets for ion-selective sensing was reported in [63]. The control system can control the droplet actuation, mixing and speed, and monitor the position until it reaches the designated detection position to accomplish selective NH_4_^+^ detection under a concentration range of 10^−6^ to 1 M, which shows the potential of the controllable and portable sensors for multiple biosensing. Coating the permeable porous polymer on the electrode array is an excellent strategy for high-throughput and addressable open-channel droplet detection (Fig. 4d) [64]. Via UV photolithography and physical vapor deposition, orthorhombic contact pads, including 20 working electrodes and 20 counter electrodes (which form a loop with working electrodes to ensure the reaction occurs on working electrodes and one pseudo-reference electrode, providing the standard potential for working electrodes) were integrated on one chip to form 400 intersection regions. Integrated circuits could be used to measure a single droplet or high-throughput 400 droplets for simultaneous detection. The performance of electrochemical sensing in droplets arrays in terms of individual addressability, reproducibility, stability and reusability, has been evaluated and affirmed, creating the possibility of high-throughput cell monitoring, drug screening and other biochemical applications.

Open-channel droplets integrated on an electrochemical platform can solve the problem of accommodating large quantities of electrode units in one chip without a short and open circuit, and promote rapid independent droplet electrochemical detection (∼100 mV/s, real-time monitoring) with a detection limit of ∼10^−17^ M [65]. Open-channel droplets as an electrochemical chamber opens the door to combining the high-throughput biosensing of trace samples with the sequential electrochemical readout of individual droplets, showing the underlying potential for real-time monitoring of multiple physiological indicators, as well as accurate disease detection.

### Droplet management for colorimetric detection

Colorimetric sensing pertains to the change of color of a solution during a chemical reaction, and color depth can be related to the concentration of the analyte in the reaction [66]. The miniaturization concept has raised the profile of the colorimetric method for high-throughput and portable sensing over the last two decades [67,68]. Particularly, a droplet array, as the typical miniaturization unit, is accessible, does not require sophisticated instruments, and is low-cost with regard to carrying out multiple qualitative, semi-quantitative and fully quantitative colorimetric sensing experiments [69].

**Figure 5.**
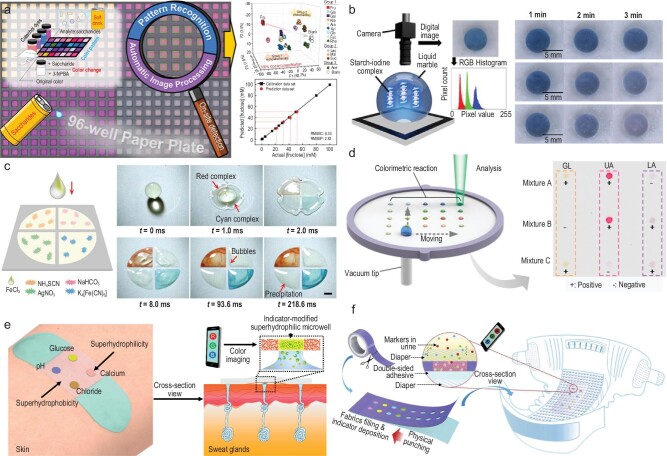
Droplet management for colorimetric detection. (a) 96-well microtiter array for detection of saccharides. Reproduced with permission from ref. [70]. (b) Liquid marbles for colorimetric enzymatic process monitoring. Reproduced with permission from ref. [71]. Copyright 2021 Wiley-VCH Verlag GmbH & Co. KGaA, Weinheim. (c) Droplet precise self-splitting for simultaneous multi-detection. Reproduced with permission from ref. [72]. Copyright 2020 Wiley-VCH Verlag GmbH & Co. KGaA, Weinheim. (d) Open-channel droplet manipulation for multiplex bioassay. Reproduced with permission from ref. [73]. Copyright 2018 American Chemical Society. (e) Superwettable and flexible bands toward sweat sampling and monitoring. Reproduced with permission from ref. [74]. Copyright 2019 American Chemical Society. Copyright 2021 American Chemical Society. (f) Microchamber bandages toward multiplexed rapid urinalysis. Reproduced with permission from ref. [75]. Copyright 2020 Elsevier.

Static droplets were originally studied for colorimetric analysis. A paper-based 96-well microtiter plate achieves the classification of 12 saccharides and semiquantification by colorimetric pattern recognition and automated algorithma, as demonstrated in Fig. 5a [70]. Nguyen *et al.* utilize the silica nanoparticle to fabricate liquid marbles for colorimetric monitoring of enzymatic processes, as demonstrated in Fig. 5b [71]. The silica nanoparticles have high light transmittance compared with other related microparticles, allowing the liquid marble to realize digital imaging-based colorimetry detection. Subsequently, some reports have focused on droplet manipulation for dynamical and sequential colorimetric sensing. The droplets go through the liquid film formation, spreading and recoiling on the surface in 7 ms to spontaneously separate hydrophilic spots at the high patterned-adhesive chip. Benefiting from no matter exchange between distributed droplets, the chip separates one droplet into five parts to achieve individual and simultaneous multiple heavy-metal-ion colorimetric analyses, which guide the use of open-channel droplets in the arenas of early diagnosis, national security and ecological monitoring (Fig. 5c) [72]. Furthermore, the combination of the vacuum tip and the superwettable substrate can effectively control the transportation and mixing of open-channel droplets at the designated location for colorimetric sensing, as shown in Fig. 5d [73]. The vacuum tip beneath the substrate pulls the droplet through a predefined area modified by the enzyme and colorimetric reagents. Some of the droplets are intercepted and continue to the next detection site. Diabetic and healthy mice are successfully determined by glucose colorimetric analysis of a plasma droplet sample, which suggests the broad applications of such a method for low-cost and rapid multiple disease diagnoses.

The integration of a colorimetric array in wearable devices is helpful for the real-time monitoring of physiological indicators. He *et al.* demonstrated a flexible colorimetric band as a wearable device that analyzes sweat *in situ*, as shown in Fig. 5e [74]. The band was made of roll-to-roll nanodendritic silica coating and oxygen plasma etching, which collected the *in situ* sweat in hydrophilic wells to react with a prestored detection reagent. A cell phone provided a readout of the % RGB of different sites to complete the real-time monitoring of physiological indicators (pH, chloride, glucose and calcium) in sweat. This has great potential for personalized medicine. Similarly, a colorimetric tape has been reported to be a user-friendly ‘just urinate’ method of urine analysis for those with urinary incontinence, and infants, as shown in Fig. 5f [75]. In contrast to superwettable substrate, this tape is physically punched to form a pattern and utilizes the height difference between the microwell and outer wall to limit droplet diffusion. A smartphone detected the multiplexed markers, including glucose, nitrite, protein and phenylpyruvate, by RGB data analysis, which brings considerable benefits to the monitoring of inconvenient biofluids.

Droplet-based colorimetric detection has been developed to diversify biosensing integrated with cameras, scanners and smartphones. Hundreds of sensing results can be read out from the high-throughput droplets at ∼1 s, and evaporation enrichment further increases the sensitivity to 10^−9^ M. However, the color difference of different angles, light reflection and refraction in image acquisition brings errors to colorimetric results, which greatly limits the quantitative analysis of droplet-based colorimetric detection when it comes to universalization and commercialization. Further efforts focus on developing a unified and standardized testing process to expand the droplet-based colorimetric method for point-of-care testing.

### Open-channel droplet SERS signal acquisition

Surface-enhanced Raman scattering, which greatly enhances normal Raman signals by enhancing the electromagnetic field on the hot spots of the noble metal substrate, has the ability to recognize chemical fingerprints of trace analytes for drug monitoring, biomarker detection and cell imaging [76]. Open-channel droplets can realize the physical settlement, enrichment and self-assembly of different noble metal particles on designated spots to form miniaturized SERS substrates for biosensing [77,78]. Concurrently, the evaporation and stirring of droplets on designated SERS sites improves the collision of the analyte with the substrate and enriches the trace targets on the SERS-active surface, resulting in highly sensitive biomolecular recognition [78,79]. The following section will discuss in detail the open-channel droplets for substrate self-assembly and trace analyte enrichment for SERS biosensing.

**Figure 6.**
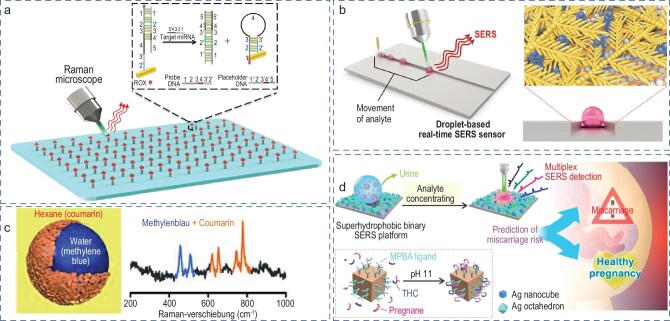
Open-channel droplet SERS signal acquisition. (a) High-throughput droplet array for breast-cancer-marker sensing. Reproduced with permission from ref. [80]. (b) Droplet-guiding platform toward real-time SERS detection. Reproduced with permission from ref. [24]. Copyright 2017 Wiley-VCH Verlag GmbH & Co. KGaA, Weinheim. (c) Liquid marbles for multiple molecular detection. Reproduced with permission from ref. [81]. Copyright 2014 Wiley-VCH Verlag GmbH & Co. KGaA, Weinheim. (d) The SERS diagnostic platform for urine metabolite sensing. Reproduced with permission from ref. [82]. Copyright 2020 American Chemical Society. Copyright 2020 Elsevier.

The hot spots of droplet-based SERS sensing are generally constructed in three ways: self-assembly of novel metal particles in droplets, construction of SERS-active substrate and collaborative enhancement of substrate and droplet. As demonstrated in Fig. 6a, the self-assembly of gold nanorods during droplet evaporation on mini-pillars builds a SERS-active surface to achieve high-throughput strand-displacement microRNA (miRNA) SERS sensing for breast cancer biomarker detection [80]. In [24], the galvanic displacement reaction on the superhydrophobic Cu plate fabricates nanodendritic silver for sequential open-channel droplet SERS analysis in Fig. 6b. Such a strategy accomplishes 100 consecutive droplet manipulations and multiple real-time rhodamine 6G SERS detection, demonstrating a potential application in automatic testing equipment. Plasmonic liquid marbles, as the natural SERS substrate, provide a new strategy for multiple-simultaneous-target SERS detection across separate organic and inorganic phases. The dense and rough Ag layer provides enough hot spots for SERS detection, and also avoids the diffusion of aqueous solution in the organic phase (Fig. 6c) [81]. The collaborative enhancement of substrate and droplet effectively improve the Raman enhancement factor. As shown in Fig. 6d, the SERS-active substrate is fabricated by the electrostatic assembly of Ag nanocubes and octahedra with a Raman enhancement factor of 2.4 × 10^9^, and the functionalized Ag nanocubes act as a new SERS substrate in droplets and can specifically capture the target molecule and stack it in a small area with high hot spot density. The combination of two SERS spots creates a ‘mirror effect’, which boosts the enhancement factor to 2.4 × 10^12^. The coupling of parts-per-trillion-level SERS results and chemometrics, including principal component analysis and partial least-squares regression, accomplishes the metabolic analysis and risk assessment of 40 patients presenting with symptoms of miscarriage [82].

The combination of open-channel droplet evaporation enrichment, substrate material structure and high-Raman-enhancing performance has raised the sensitivity of Raman detection to the level of single-molecule detection, providing wide application prospects. This is a simple, accessible and portable strategy. The regrettable drawback is that the heterogeneity of substrate structure and droplet dispersion causes extremely low reproducibility for quantitative analysis. Future studies should further homogenize the structure and reduce the droplet volume so that the detection spot matches the laser diameter, to facilitate the droplet-based SERS method in practical and commercial quantitative analysis.

### Open-channel droplet array for mass spectrometry

**Figure 7.**
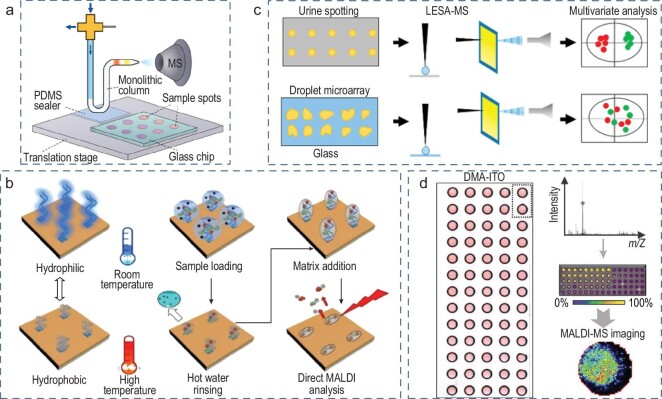
Open-channel droplet array for mass spectrometry. (a) Swan-shaped MS probe for sample separation and analysis. Reproduced with permission from ref. [84]. Copyright 2020 American Chemical Society. (b) Smart MALDI-MS plate for protein purification and analysis. Reproduced with permission from ref. [85]. Copyright 2018 American Chemical Society. (c) Droplet microarray toward liquid extraction surface MS analysis. Reproduced with permission from ref. [86]. Copyright 2018 American Chemical Society. (d) MS imaging of nanoliter-scale cell assays. Reproduced with permission from ref. [87]. Copyright 2021 Wiley-VCH Verlag GmbH & Co. KGaA, Weinheim.

Mass spectrometry (MS) analysis in biochemistry and biology has expanded over the past few decades to cover many aspects of structural and molecular biology [83]. The open-channel droplet array contributes to uniform sample preparation, desalination and purification during MS, and improves the sensitivity (10^−12^ M) and accuracy of biosensing, with low relative standard deviation (8%). A high-throughput open-channel sample went through desalting analysis via the liquid chromatography separation-MS device in [84]. The poly(N-isopropylacrylamide) (PNIPAM)-modified thermos-responsive open-channel-droplet matrix-assisted laser desorption/ionization (MALDI) plate was used for enrichment, desalting and MS analysis (Fig. 7b) [85]. Furthermore, the droplet microarray confining the sample spot can effectively improve extraction repeatability and spectral reproducibility. As presented in Fig. 7c, the droplet microarray for urine sample analysis shows a low relative standard deviation below 8%, and glass extraction results in relative standard deviations (RSDs) are ∼20% over the acceptable precision (15%) [86]. Furthermore, the open-channel droplet array with MS can focus on high-demand cell analysis. MALDI MS imaging (MALDI–MSI) has successfully achieved cell-based pharmaceutical compound profiling, as shown in Fig. 7d [87]. A superwettable array was integrated on indium tin oxide (ITO) substrate as a cell incubator for optical microscopy and MS readouts. During the A549 lung cancer cell culture in the microwells, the introduction of GSK2194069, a selective fatty acid synthase inhibitor, promoted the intracellular accumulation of fatty acid synthase (FASN), allowing MALDI–MS to determine the influence of concentration on the inhibition process through ion intensity imaging of a superwettable array.

### Other sensing strategies

In addition to the above traditional testing methods, some new strategies were exploited to collect the information in open-channel droplets [88,89]. For example, Gao *et al.* implemented a pH-responsive platform to detect the pH, urea and glucose in human saliva and urine by measuring open-channel droplet contact angles on a superwetting surface [90]. Similarly, the critical sliding angle can be employed as the quantitative index for visualized trace target sensing in open-channel droplets [25]. The combination of rolling circle amplification (RCA) reactions with DNA-catalyzed strand displacement reactions controls the DNA length to regulate the droplet movement for achieving signal reading of small molecule, nucleic acid and protein, indicating great potential for multiple targets of cells and serums sensing. Nowadays, various emerging strategies are continuously being developed to easily and quickly extract key information about physiological health or biochemical reactions contained in open-channel droplets.

### Multi-technology coupling toward droplet analysis

Open-channel droplets are prone to signal deviation caused by environmental fluctuations, and the output of a single signal has difficultly satisfying the high demand for biochemical analysis. For this reason, multifunctional integration is developed to realize simultaneous reading of two or more signals in the same open-channel droplets [39,91]. Such a multi-signal strategy can not only couple multiple signals in order to eliminate the false position and improve detection accuracy, but also aid mutual-coupling in-depth mechanism research.

**Figure 8.**
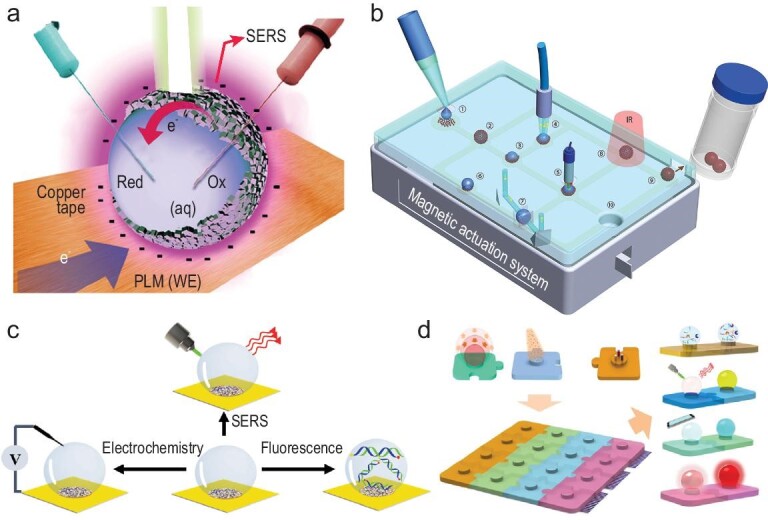
Multi-technology coupling toward biosensing in droplets. (a) 3D plasmonic liquid marble for molecular-level spectroelectrochemical investigation. Reproduced with permission from ref. [13]. Copyright 2017 Wiley-VCH Verlag GmbH & Co. KGaA, Weinheim. (b) ‘On-line’ detection based on magnetic liquid marble. Reproduced with permission from ref. [39]. Copyright 2015 Wiley-VCH Verlag GmbH & Co. KGaA, Weinheim. (c) Nanodendritic gold/graphene-based droplet biosensor for tri-modal miRNA sensing. Reproduced with permission from ref. [91]. Copyright 2019 The Royal Society of Chemistry. (d) Jigsaw-like mini-pillar platform for multi-mode biosensing. Reproduced with permission from ref. [92]. Copyright 2021 Elsevier.

Plasmonic liquid marble as the 3D working electrode and SERS substrate achieves dual-signal molecular-level spectra electrochemical sensing. The coupling of the SERS spectrum and electrical signals demonstrates the immense potential for real-time molecular-level identification of specified targets and provides molecular-level identification and quantification of the entire electron-transfer process (Fig. 8a). Similarly, the magnetic liquid marbles, as an ‘on-line’ laboratory, are employed for electrochemical and optical sensing, as shown in Fig. 8b [39]. Attempts have been made to integrate more modal signals into a droplet reaction platform for accurate and sensitive sensing. Song *et al.* integrated nanodendritic gold with graphene in superwettable microwells for fluorescence, SERS and electrochemical tri-mode sensing, as shown in Fig. 8c [91]. Tri-mode signal coupling greatly improves the accuracy of trace sensing and demonstrates potential applications in precise early diagnosis and real-time monitoring. Subsequently, Song *et al.* utilize the jigsaw-like mini-pillar platform for electrochemical, fluorescence, SERS and colorimetric sensing in open-channel droplets (Fig. 8d) [92]. Each mini-pillar as an individual reactor can achieve one specified detection by specified modification and has a specific concave-convex interface to joint with others, forming a multiple sensing platform.

Multi-signal coupling is an inevitable trend in the development of open-channel biosensing, and reduces the errors caused by aberrant fluctuation, improving the accuracy of monitoring. However, the balancing of size and cost of the open-channel platform with multi-signal ‘all-in-one’ integration is an insurmountable problem. It is expected that the next generation of droplet-based platforms will be integrated, multifunctional and portable for complicated and laborious biosensing.

## CONCLUSIONS AND OUTLOOK

In conclusion, we summarized recent progress with regard to diversified open-channel droplet arrays being utilized as versatile biosensing reactors, discussing the classification of the open-channel platform (droplet under surface, such as microchamber; droplet at surface, such as wettable pattern; droplet on surface, such as mini-pillar platform; droplet in surface, such as liquid marbles), the construction approach, potential applications and inherent limitations. We focused on the open-channel platform combined with multiple signal transmission strategies (electrochemical, SERS, colorimetric, fluorescence, MS and multiple signal coupling) for tracing and sensing of inorganic matter, nucleic acid, proteins, cells, etc. With increasing demand and technological innovation, more diverse platforms and sensing approaches will be applied to open-channel droplet biosensing. However, there still remain several scientific and technological challenges from basic through to applied research (Table 2).

**Table 2.** Summary of the advantages, disadvantages and challenges of open-channel droplets for biosensing.

Main advantages	Limitations	Challenges
High sensitivity Larger specific surface Low sample consumption High-throughput analysis Accelerated reactions Accessible purification and mixing Accessible multiple device integration	Uncontrollable evaporation Irresistible contact and residue Missing multiple factors integration High-throughput signal interference	Construct stabilized and controllable microsystems Develop intelligent signal acquisition and processing technologies Controllable evaporation Construct contact-free droplet array

### Instability of the microsystem

A stable signal output is an essential factor for the detection of reproducibility and continuous monitoring. Open-channel droplets need to solve the serious problem of providing steady-state conditions. Firstly, open-channel platforms only anchor the droplets so that they do not fall, but cannot precisely control shaking, vibration and deformation. For example, the platform from 0° inverted to 180° when the sphere of droplets changed to an ellipse, causing the signal fluctuation. Secondly, a large specific surface area accelerates droplet interaction with the atmosphere, resulting in susceptibility to environmental fluctuations. A small variation in temperature, humidity or wind speed may lead to a fundamentally tremendous change in an internal system, like the ‘butterfly effect’. Thus, both factors restricted the open-channel droplet array from making the leap from laboratory to practical application, and future efforts will focus on improving the microsystem’s resistance to such handicaps and achieving accurate and reproducible multiple signal acquisition.

### Intelligent signal output

The output results being comprehensive and clear means that the open-channel sensing system can be used in a wide range of scenarios by a wide range of users. Current data results, including electrochemical curves, Raman diagrams and fluorescence imaging, require professional comparison and analysis in order to obtain the required detection results, which greatly limits the application of such sensors in the commercial sphere. Some studies have attempted to transmit the signal to the terminal and use relevant software to transform the signal into visual results [93,94]. However, such a strategy only deals with the signal but ignores the influence of the environment and other factors on the signal results. Future intelligent signal output will need to integrate data results with external factors, including temperature, humidity and vibration, to produce the most realistic physiological results.

### Controllable evaporation

Evaporation of open-channel droplets may bring uncontrollable fluctuation to detection results, which is an undesired side effect of the reaction process and a desired side effect of the sensing process. Controllable evaporation can effectively improve the accuracy and sensitivity of biosensing. Accelerated evaporation can be controlled by integrating thermally responsive materials in droplets or the substrate, which heat up in a short period of time, resulting in analysis enrichment. Furthermore, ultrasonic, electric and other types of stimulation achieve the rapid mixing and dispersion of droplets, resulting in controllable accelerated evaporation. To control undesired evaporation, the conventional method is to transfer the droplet platform in a closed constant humidity or microthermal space. A future strategy could be to develop an intelligent system to monitor the volume loss of droplets, accurately supplementing the lost volume to achieve a dynamic balance of the concentration of various analytes in the droplets to effectively solve the evaporation problem.

### Contact-free droplet array

At present, most open-channel arrays rely on the platform interface, and unnecessary contact between the sample and surface is inevitable. For ultratrace-droplet or single-molecule-level sensing, such contact and residue cause the signal to go missing. Some researchers have reported the suspending of droplets by sound field or magnetic field, for mechanical studying and droplet dynamics investigation [95,96]. Such a contact-free strategy, catering to the general trend of open-channel droplet sensing, not only improves the accuracy of ultratrace detection but completely gets rid of the interface to the droplet limitation to motion, fusion and splitting in three-dimensional space. A future direction would be to try to combine suspension droplets with diversified detection technologies and maintain the existing sensitivity in the absence of substrate signal enhancement.

Although a number of unsolved critical issues should be addressed, the advantages of low-cost, high throughput, controllability and an integrated open-channel have encouraged the scientific community to devote more effort to accelerating comprehensive and commercial development. In the future, the open-channel droplet platform will be prominent in the field of comprehensive biosensing for exudative body fluid analysis to invasive tissue fluid/blood detection, with its advantages of stability, portability and integration. We hope that this review will help researchers understand current emerging open-channel droplets for biosensing and select the appropriate droplets platform and technology to complete the required biosensing, leading to continuing scientific and industrial advances.
